# May the clamp be with you

**DOI:** 10.1093/icvts/ivad147

**Published:** 2023-08-31

**Authors:** Lorenzo Bragaglia, Cecilia Menna, Mohsen Ibrahim

**Affiliations:** Division of Thoracic Surgery, Department of Medical and Surgical Sciences and Translational Medicine, Sapienza University of Rome, Sant'Andrea Hospital, Rome, Italy; Division of Thoracic Surgery, Department of Medical and Surgical Sciences and Translational Medicine, Sapienza University of Rome, Sant'Andrea Hospital, Rome, Italy; Division of Thoracic Surgery, Department of Medical and Surgical Sciences and Translational Medicine, Sapienza University of Rome, Sant'Andrea Hospital, Rome, Italy

**Keywords:** Thoracoscopic surgery, Conversion rate, Clamping technique, Main pulmonary artery

The use of video-assisted thoracoscopic surgery has been exponentially increasing in the last few decades and >30% of lobectomy for lung cancer today are performed using minimally invasive techniques [1]. For this reason, nowadays, surgeons’ efforts should be guided towards improving surgical techniques, especially to reinforce the role of minimally invasive surgery, even in complicated thoracoscopic settings.

In the literature, conversion rates for video-assisted thoracoscopic to open surgery range between 2% and 23%. Although oncological reasons are the most common causes of conversion, intraoperative complications frequently lead to conversion to thoracotomy. Bleeding is one of the most terrifying and challenging complications to deal with [2]. Bleeding from vascular injury, usually bleeding from branches of the pulmonary artery, can occur in thoracoscopic surgery. Clamping the pulmonary artery is necessary for arterial repair. Less frequently, clamping the pulmonary artery is intended for thoracoscopic arterial sleeve resection [3].

This study investigates different feasible clamping techniques in Thoracoscopic anatomical lung resection: the double-loop technique, DeBakey clamp, Fogarty clamp, endovascular clips and vessel loop technique. Chiba *et al.* [4] specifically focused on evaluating the efficacy and safety of the double-loop technique for pulmonary arterioplasty and/or injured artery repair. The study is divided into 2 main different parts: an experimental study in which a main pulmonary artery model has been used to evaluate the pressure resistance capacity and burst pressure of each clamping technique investigated, and a histological study aimed to evaluate the intimal damage of the human pulmonary artery associated with double loop technique and DeBakey clamp.

According to the results of the experimental study, the double loop technique and DeBakey clamping at the third notch seem to be not significantly different in measured burst pressure and clamp pressure.

Actually, investigating the intimal damage of clamping techniques in histological examination, the double-loop technique resulted in only intimal deformation in major part of the samples. Moreover, the double loop technique demonstrated advantages, such as an unbarred thoracoscopic view, compared to other techniques. Chiba *et al.* [4] demonstrated that the double loop technique is feasible and safe in video-assisted thoracoscopic arterioplasty and injured artery repair.

This is the first study that evaluates the efficacy and safety of the available clamping technique in thoracoscopic anatomical lung resection using a main pulmonary artery model. The remarkable technology used during this study is, undoubtedly, one of its strengths.

However, the use restricted to cadavers or human specimens undergoing pneumonectomy, lacking in potential histological different evidences showed for *in vivo* scenarios, could represent a bias. Histological changes due to formalin fixation in cadaver’s specimen should be considered. Moreover, it is clear that enrolling human cases undergoing pneumonectomy to obtain adequate specimens could be challenging due to the increasing of early-stage lung cancer diagnosis.

The control of bleeding in thoracoscopic surgery can be obtained through different techniques, such as electrocautery, compression with gauze, local haemostatics or clamping the upstream principal vessel [5]. The latter often consists in localizing the site of bleeding, completing the pulmonary artery dissection and isolation, to control the haemorrhage. In an emergency context, especially for less experienced surgeons, the conversion to open approach remains the safest choice. In these cases, it is important to maintain the control of the surgical field and, at the same time, to act promptly [6]. For this reason, the double loop technique, despite its efficacy, could present a disadvantage in terms of time in emergency and instable situations.

On the other hand, the double loop technique may offer its advantages in preventing bleedings when performed during arterioplasty and not in bleeding circumstances. In pulmonary artery thoracoscopic resections, in fact, especially in cases with a preoperative high risk of bleeding or vessels injuries, this technique may offer interesting results. Furthermore, in this setting, even the use of bulldogs or silk-suture or silicone catheter tourniquets may show the same advantages of easy application ensuring an optimal thoracoscopic view [7].

Certainly, histological and mechanistic results are interesting and propaedeutic to further clinical studies. It would be noteworthy to understand whether could sustain potential higher rates of cerebral and vascular accident, death or dehiscence in the postoperative period in *in vivo* investigation.
